# A Modulated Wideband Converter Model Based on Linear Algebra and Its Application to Fast Calibration

**DOI:** 10.3390/s22197381

**Published:** 2022-09-28

**Authors:** Gilles Burel, Anthony Fiche, Roland Gautier

**Affiliations:** Université de Bretagne Occidentale, Lab-STICC, CNRS, UMR 6285, 6 avenue Le Gorgeu, 29200 Brest, France

**Keywords:** compressed sampling, hardware calibration, spectrum monitoring, linear algebra, matrix theory, modulated wideband converter

## Abstract

In the context of cognitive radio, smart cities and Internet-of-Things, the need for advanced radio spectrum monitoring becomes crucial. However, surveillance of a wide frequency band without using extremely expensive high sampling rate devices is a challenging task. The recent development of compressed sampling approaches offers a promising solution to these problems. In this context, the Modulated Wideband Converter (MWC), a blind sub-Nyquist sampling system, is probably the most realistic approach and was successfully validated in real-world conditions. The MWC can be realized with existing analog components, and there exist calibration methods that are able to integrate the imperfections of the mixers, filters and ADCs, hence allowing its use in the real world. The MWC underlying model is based on signal processing concepts such as filtering, modulation, Fourier series decomposition, oversampling and undersampling, spectrum aliasing, and so on, as well as in-flow data processing. In this paper, we develop an MWC model that is entirely based on linear algebra, matrix theory and block processing. We show that this approach has many interests: straightforward translation of mathematical equations into simple and efficient software programming, suppression of some constraints of the initial model, and providing a basis for the development of an extremely fast system calibration method. With a typical MWC acquisition device, we obtained a speed-up of the calibration computation time by a factor greater than 20 compared with a previous implementation.

## 1. Introduction

Digital wireless radio signals are often composed of a small number of narrow-band transmissions spread across a wide spectrum range. For example, the Internet-of-Things (IoT) communications have recently emerged in contexts such as smart cities. Cognitive radios are able to manage the spectrum dynamically but require advanced sensing techniques for spectrum monitoring.

Basically, spectrum monitoring is based on the Shannon–Nyquist sampling theorem [1,2]. This theorem states that the signal must be sampled at a rate greater than its Nyquist frequency, which is twice its frequency band. However, when we have to monitor a large frequency band, this requirement can exceed the capabilities of existing Analog to Digital Converters (ADC) or require very expensive components. Furthermore, sampling at a very high rate may require huge storage capacities to store the digital samples.

Recently, new approaches have been proposed allowing sampling at sub-Nyquist rates. These approaches, known as compressed sensing, or compressive sampling [3], have emerged as a promising framework for signal acquisition in difficult applications, such as monitoring a wideband spectrum [4]. The basic idea of compressed sampling is to take advantage of the fact that a signal that has a sparse representation on a given basis can theoretically be recovered from a small set of linear measurements [5,6]. The price to pay is the need to develop sophisticated signal processing algorithms to reconstruct the signal from this small set of measurements, these algorithms being much more complex than the usual demodulators.

A great deal of the theoretical aspects of compressed sampling has been addressed in the literature. For example, many studies have been proposed in relation to the design of the measurement scheme as in [7,8]. However, few studies have considered the practical limitations of compressed acquisition. In fact, designing measurement schemes and applying them to practical acquisition systems remains a significant challenge.

In this context, the Modulated Wideband Converter (MWC) has been proposed as an efficient system for real-world compressed sampling [9]. The MWC does sub-Nyquist sampling without prior information about the spectral support of the transmitters present in the monitored wideband. It can be realized with existing devices [10] and has been successfully tested on real-world problems, including surveillance of a wideband spectrum [11].

A few real-world compressed sensing acquisition systems have already been proposed [11,12,13,14,15,16,17,18]. An analog circuit board with discrete components was designed by us to prototype compressed sensing based on the MWC principle [19].

A necessary step when using MWC in real-world conditions is the calibration of the acquisition system. Indeed, analog components are never ideal, especially when fed with wideband signals. Then, using a purely theoretical model leads to extremely poor performances of signal reconstruction. An efficient calibration method, which is considered a reference, has been proposed in [13]. It consists of estimating the sensing matrix column after column by injecting sine waves at a specific frequency and recording the corresponding output signals. The procedure is repeated by changing the input frequency until all columns are estimated. Some researchers have exploited this work to calibrate their systems or to propose variants of the calibration algorithms [18,20,21,22,23]. While this procedure is efficient, it can be time-consuming because the number of columns to estimate is usually at least a few dozen. That is why a few authors [24,25], including us [19], have recently proposed alternative calibration algorithms requiring only one input signal.

The MWC theoretical background is signal processing theory (filters, modulation, Fourier series, sampling theory, spectrum aliasing, etc.). Most signal processing theoretical tools are asymptotic. However, when signals are processed in real-world conditions, they are always finite; thus, block processing and purely matrix-based algorithms may be more natural and efficient.

Moreover, a quick look at the literature shows that most people use Matrix-based programming tools, such as Octave or Matlab, for signal processing in this context, but without really exploiting the power of these tools. To take full advantage of the power of Matrix-oriented software, it would then be preferable to process data by blocks instead of in-flow. It is, therefore, interesting to view the whole MWC acquisition and reconstruction in terms of block processing. The most natural framework to achieve this objective is matrix theory and linear algebra.

In this paper, we elaborate on an MWC model using linear algebra only (without any signal processing theory). While this approach will probably appear less intuitive than the approach based on signal processing, because most people are less familiar with linear algebra than with signal processing, it has strong advantages:

- Once the model is established, programming it becomes extremely simple, straightforward and efficient.
- Furthermore, computational complexity is significantly reduced.
- The border effects are implicitly taken into account in the model. Indeed, using a signal processing model, people have to deal with the fact that the signals processed in the real world are not infinite, while when using a linear algebra model, the finite nature of the data is implicitly taken into account and the mathematical equations are exact and not approximate.
- In the original version of the MWC, the number of physical branches is increased by a factor *q*, which must be an odd integer due to signal processing considerations. An interesting aspect of the linear algebra model is that it allows even integers for *q*.

The main contributions of this paper are:

- The development of a pure linear-algebra model of the MWC. Despite the fact that establishing this model is rather hard because it requires non-trivial matrix manipulations, once established, it is extremely simple and allows programming MWC-related software, such as calibration, in a very fast, compact and efficient way.
- Its application to the development of a very fast calibration method. With typical choices of parameters, the calibration is more than 20 times faster than our previous method (this previous method being itself very fast compared to a reference method because it required only one calibration signal instead of dozens of sinusoidal signals in the reference method).

The remainder of this paper is organized as follows: Section 2 provides the main mathematical tools used in the paper. Then, Section 3 presents an overview of our hardware acquisition board and the MWC principle. Section 4 establishes a system model based on linear algebra, and an equivalent model, useful for signal reconstruction and system calibration, is then derived in Section 5. In Section 6, we show how this model allows us to considerably improve a calibration method that we proposed previously, leading to speeding up the process by a factor greater than 20. Then, some experimental results are shown in Section 7.

## 2. Mathematical Background

### 2.1. Notations

Unless otherwise stated, lowercase symbols denote row vectors (e.g., *x*, *p*), uppercase symbols denote matrices (e.g., *C*, *Z*), $\bar{x}$ stands for the DFT (Discrete Fourier Transform) of *x*. The symbols N,K,L,a,b will be used to denote the size of vectors or matrices.

We will denote $D_{x}$ as the square diagonal matrix whose diagonal is vector *x*.

The vectorization of a K×L matrix *Q*, denoted vec(Q), is the 1×KL row vector obtained by reading the matrix row after row, from top to bottom:

(1) $$vec\left(Q\right)=\left(\begin{array}{ccc} q_{11}\cdots q_{1L} & q_{21}\cdots q_{2L} & \cdots \;q_{K1}\cdots q_{KL} \end{array}\right)$$

$M^{*}$ stands for the Hermitian transpose of matrix M.

$I_{K}$ stands for the K×K identity matrix.

The nearest lower or equal integer will be noted ⌊ ⌋ and the nearest greater or equal integer ⌈ ⌉.

### 2.2. Circulant Matrices

Let *x* be a 1×N row vector. A circulant matrix $C_{x}$ is a square matrix whose first row is *x* and each next row is a circular shift one element to the right of the preceding row. That is:

(2) $$C_{x}=\left(\begin{array}{ccccc} x_{o} & x_{1} & x_{2} & \cdots & x_{N-1}\\ x_{N-1} & x_{0} & x_{1} & \cdots & x_{N-2}\\ x_{N-2} & x_{N-1} & x_{0} & \cdots & x_{N-3}\\ \vdots & \vdots & \vdots & \ddots & \vdots \\ x_{1} & x_{2} & x_{3} & \cdots & x_{0} \end{array}\right)$$

It is convenient to define the cyclic permutation matrix as the N×N matrix below:

(3) $$J_{N}=\left(\begin{array}{ccccc} 0 & 1 & 0 & \cdots & 0\\ 0 & 0 & 1 & \; & 0\\ \vdots & \; & & \ddots \\ 0 & 0 & 0 & \; & 1\\ 1 & 0 & 0 & \cdots & 0 \end{array}\right)$$

Then, $C_{x}$ is a polynomial in $J_{N}$:

(4) $$C_{x}=\sum_{n=0}^{N-1}x_{n}J_{N}^{n}$$

The effect of multiplication of a matrix *M* by $J_{N}$ is as follows. The rows of $MJ_{N}$ are the rows of *M* circularly shifted one element to the right. The columns of $J_{N}M$ are the columns of *M* circularly shifted one element to the top.

Matrices $J_{N}$ and $C_{x}$ commute:

(5) $$J_{N}C_{x}=C_{x}J_{N}$$

because

(6) $$\begin{array}{cc} J_{N}C_{x} & =J_{N}\left(\sum_{n=0}^{N-1}x_{n}J_{N}^{n}\right) \end{array}$$

(7) $$\begin{array}{cc} \; & =\sum_{n=0}^{N-1}x_{n}J_{N}^{n+1} \end{array}$$

(8) $$\begin{array}{cc} \; & =\left(\sum_{n=0}^{N-1}x_{n}J_{N}^{n}\right)J_{N} \end{array}$$

(9) $$\begin{array}{cc} \; & =C_{x}J_{N} \end{array}$$

### 2.3. Discrete Fourier Transform (DFT)

Let us note ω the $N^{th}$ square root of unity below:

(10) $$\omega =\exp\left(-i\frac{2\pi }{N}\right)$$

The DFT matrix $F_{N}$ is an N×N square symmetric matrix whose element at row *l* column *k* is $\omega^{lk}$ (assuming row 0 is the first row, and column 0 the first column):

(11) $$F_{N}=\left(\begin{array}{ccccc} 1 & 1 & 1 & \cdots & 1\\ 1 & \omega & \omega^{2} & \cdots & \omega^{N-1}\\ 1 & \omega^{2} & \omega^{4} & \cdots & \omega^{2(N-1)}\\ \vdots & \vdots & \vdots & \ddots & \vdots \\ 1 & \omega^{N-1} & \omega^{2(N-1)} & \cdots & \omega^{{\left(N-1\right)}^{2}} \end{array}\right)$$

The inverse DFT matrix is

(12) $$F_{N}^{-1}=\frac{1}{N}F_{N}^{*}$$

The DFT of a vector *x* is

(13) $$\bar{x}=xF_{N}$$

and the inverse DFT (IDFT) is given by $\bar{x}F_{N}^{-1}$.

A circulant matrix is diagonalized by the DFT matrix. That is

(14) $$C_{x}=F_{N}D_{\bar{x}}F_{N}^{-1}$$

It follows that the elements of $\bar{x}$ are the eigenvalues of $C_{x}$ and the columns of $F_{N}^{-1}$ are the eigenvectors.

We also have

(15) $$D_{\bar{x}}=F_{N}^{-1}C_{x}F_{N}$$

and

(16) $$\frac{1}{N}C_{\bar{x}}=F_{N}^{-1}D_{x}F_{N}.$$

### 2.4. Kronecker Product

The Kronecker product is a bilinear matrix operation, denoted by ⊗. If *A* is a K×L matrix and *B* is a M×N matrix, it produces the KM×LN block matrix *C* below:

(17) $$C=A⊗B=\left(\begin{array}{ccc} a_{11}B & \cdots & a_{1L}B\\ \vdots & \ddots & \vdots \\ a_{K1}B & \cdots & a_{KL}B \end{array}\right)$$

A useful property about the inverse is:

(18) $${\left(A⊗B\right)}^{-1}=A^{-1}⊗B^{-1}$$

Assuming the sizes are such that one can form the matrix products AC and BD, an interesting property, known as the mixed-product property, is:

(19) (A⊗B)(C⊗D)=(AC)⊗(BD)

The product is not commutative, but there exist permutation matrices (shuffle matrices) such that if *A* is an a×a square matrix and *B* a b×b square matrix, then [26]:

(20) $$A⊗B=P_{a,b}\left(B⊗A\right)P_{b,a}$$

Matrix $P_{a,b}$ represents the permutation obtained when elements, written row by row in an a×b matrix, are read column by column. For instance, set a=2 and b=3, and write the elements 1,2,3,4,5,6 row by row in a a×b matrix

(21) $$\left(\begin{array}{ccc} 1 & 2 & 3\\ 4 & 5 & 6 \end{array}\right)$$

Reading column by column, we obtain 1,4,2,5,3,6. The permutation matrix is then the matrix such that:

(22) $$\left(\begin{array}{cccccc} 1 & 4 & 2 & 5 & 3 & 6 \end{array}\right)=\left(\begin{array}{cccccc} 1 & 2 & 3 & 4 & 5 & 6 \end{array}\right)P_{2,3}$$

That is:

(23) $$P_{2,3}=\left(\begin{array}{cccccc} 1\\ \; & \; & 1\\ \; & \; & & \; & 1\\ \; & 1\\ \; & \; & & 1\\ \; & \; & & \; & & 1 \end{array}\right)$$

If N=KL, an interesting property with the permutation matrix defined in (3) is

(24) $$J_{N}^{K}=J_{L}⊗I_{K}.$$

### 2.5. General Radix Identity

If *N* is a composite number, i.e., N=KL, then [26]:

(25) $$F_{N}=\left(F_{K}⊗I_{L}\right)T_{K,L}\left(I_{K}⊗F_{L}\right)P_{K,L}$$

where $T_{K,L}$ is a diagonal matrix (twiddle matrix) and $P_{K,L}$ a permutation matrix (shuffle matrix defined in Section 2.4).

The twiddle matrix $T_{K,L}$ is an N×N diagonal matrix, the diagonal of which is $vec(Q_{K,L})$ with ω defined in Equation (10) and

(26) $$Q_{K,L}=\left(\begin{array}{ccccc} 1 & 1 & 1 & \cdots & 1\\ 1 & \omega & \omega^{2} & \cdots & \omega^{L-1}\\ 1 & \omega^{2} & \omega^{4} & \cdots & \omega^{2(L-1)}\\ \vdots & \vdots & \vdots & \ddots & \vdots \\ 1 & \omega^{K-1} & \omega^{2(K-1)} & \cdots & \omega^{\left(K-1)(L-1\right)} \end{array}\right)$$

For instance, with K=2 and L=3, we have

(27) $$Q_{2,3}=\left(\begin{array}{ccc} 1 & 1 & 1\\ 1 & e^{-i\pi /3} & e^{-2i\pi /3} \end{array}\right)$$

and the diagonal of $T_{2,3}$ is

(28) $$diag\left(T_{2,3}\right)=\left(\begin{array}{cccccc} 1 & 1 & 1 & 1 & e^{-i\pi /3} & e^{-2i\pi /3} \end{array}\right)$$

Let us note $\theta_{K}$ and $1_{K}$ as the (1×K) vectors below

(29) $$\theta_{K}=\left[\begin{array}{cccc} 1 & 1 & \cdots & 1 \end{array}\right]$$

(30) $$1_{K}=\left[\begin{array}{cccc} 1 & 0 & \cdots & 0 \end{array}\right]$$

Note that for any 1×L vector *p*, we have

(31) $$\left(1_{K}⊗p\right)T_{K,L}=\left(1_{K}⊗p\right)$$

because only the first *L* elements of $1_{K}⊗p$ are non null, and the *L* first elements of $T_{K,L}$ are ones.

Note also that

(32) $$\left(1_{K}⊗p\right)P_{K,L}=p⊗1_{K}$$

when elements of $1_{K}⊗p$ are written row by row in a K×L matrix, the elements of *p* go on the first row and the K−1 next rows are null. Then, when this matrix is read column by column, we obtain elements of *p* separated by K−1 zeroes, that is, $p⊗1_{K}$

Similarly, it is easy to check that

(33) $$T_{a,b}^{-1}\left(I_{a}⊗1_{b}^{T}\right)=I_{a}⊗1_{b}^{T}$$

and

(34) $$P_{a,b}^{-1}\left(I_{a}⊗\theta_{b}^{T}\right)=\theta_{b}^{T}⊗I_{a}.$$

### 2.6. Selection Matrix

Let us define the selection matrix $S_{N,K}^{\left(r\right)}$ as the N×K matrix below:

(35) $$S_{N,K}^{\left(r\right)}=\left(\begin{array}{c} 0_{r\times K}\\ I_{K}\\ 0_{(N-K-r)\times K} \end{array}\right)$$

If $x=\left(x_{0}\cdots x_{N-1}\right)$ is a 1×N vector, then $y=xS_{N,K}^{\left(r\right)}$ is the 1×K vector below:

(36) $$y=\left(x_{r}\cdots x_{r+K-1}\right)$$

We consider the indices modulo *N*, so *r* may be negative.

### 2.7. Moore–Penrose Pseudo-Inverse

Let us consider a rectangular matrix *Z* whose size is L×K with L≤K. The Moore–Penrose pseudo-inverse [27] of *Z*, denoted $Z^{+}$, is a K×L matrix, which generalizes the concept of inverse and, among other interesting properties, provides a mean to compute a least squares solution to a system of linear equations that lacks an exact solution. The pseudo-inverse is defined and unique for all complex matrices. It is usually computed using the singular value decomposition (SVD).

Let us note the SVD of *Z* as [28]:

(37) $$Z=USV^{*}$$

where *U* is a L×L unitary matrix (i.e., $UU^{*}=U^{*}U=I$), *V* is a K×L matrix with orthonormal columns (i.e., $V^{*}V=I$) and *S* is a diagonal matrix whose diagonal elements are the singular values (non-negative real numbers, ranked by decreasing order). The SVD exists for all complex matrices.

Here we consider a version of the SVD usually called “thin-SVD”, which is a compact version of the more general SVD decomposition (in which matrices *S* and *V* are larger), because this compact version is sufficient for the purpose of computing the pseudo-inverse. The computational cost of computing the thin-SVD is $6KL^{2}+20L^{3}$ flops ([28] p. 254). Note that for complex matrices, it is usual to redefine the floating point operation (flop) in order to count only one flop for the product of two complex numbers, while in reality it requires four real multiplications. Since only ratios between the computational costs of algorithms are of interest, applying this does not change the result.

The pseudo-inverse is given by:

(38) $$Z^{+}=VS^{+}U^{*}$$

where $S^{+}$ is the pseudo-inverse of *S*. It is a diagonal matrix in which the diagonal contains the inverses of the singular values of *S*, which are above a small tolerance value, and 0 elsewhere.

The cost of the inversion plus the computation of the matrix product is $2L+KL^{2}≃KL^{2}$.

Overall, the cost of computing the pseudo-inverse is $7KL^{2}+20L^{3}$.

## 3. Acquisition Device and System Parameters

The MWC is a compressed sampling device that samples a signal x(t) at a sampling frequency $F_{s}$ significantly lower than its Nyquist frequency $F_{nyq}$. The input signal is assumed sparse in the frequency domain. From the outputs of this acquisition device, one can reconstruct the input signal using a compressed sensing algorithm, such as Orthogonal Matching Pursuit (OMP) [29].

**Figure 1:**
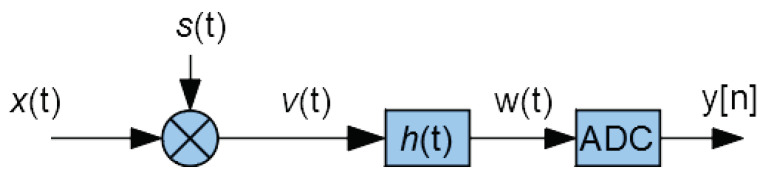
Principle of MWC acquisition (one physical branch).

The principle of the MWC is shown in Figure 1:

- The input signal x(t) is multiplied (using a mixer) by a scrambling signal s(t).
- The resulting signal v(t) goes through a low-pass filter whose impulse response is h(t).
- Then, the filter output w(t) is sampled by an Analog to Digital Converter (ADC), providing the output samples y[n].

The scrambler s(t) is a periodic signal: it is a basic waveform p(t) repeated $F_{p}$ times per second. The analog waveform p(t) itself is generated at sampling frequency $F_{nyq}$ from an *L* samples digital sequence, which is usually a pseudo-random sequence. Consequently, we have $F_{p}=F_{nyq}/L$.

The performance of the system can be enhanced by using *M* parallel branches with different scrambling signals. However, since generalization to *M* branches is trivial, we will restrict the discussions below to one branch.

The digital outputs y[n] are provided at $F_{s}$ samples per second.

In previous practical realizations, in order to reduce aliasing, the ADC output samples go through a digital filter, which provides properly filtered samples at a frequency $F_{ss}$ lower than $F_{s}$. In the original MWC model, $F_{ss}$ is an odd multiple of $F_{p}$, that is $F_{ss}=qF_{p}$ with *q* an odd integer. In this paper, since the linear algebra model allows a less constrained post-processing, this digital filter is not required and *q* is not necessarily odd. Indeed, we will see that the linear algebra model also allows even values of *q*.

When designing an actual acquisition device, we have to choose some parameters:

- The sampling frequency $F_{nyq}$ of the scramblers, which will impact the Nyquist frequency of acceptable input signals (i.e., input signal maximum frequency must remain under $F_{nyq}/2$).
- The sampling frequency $F_{s}$ of the ADC, which should be significantly lower than $F_{nyq}$ (otherwise the system would have no interest compared to direct sampling at $F_{nyq}$). This frequency determines the subsampling factor $b=F_{nyq}/F_{s}$.
- The length *L* of the scrambler periodic pattern. This parameter determines the frequency of repetition $F_{p}=F_{nyq}/L$ of the scrambling pattern.

**Figure 2:**
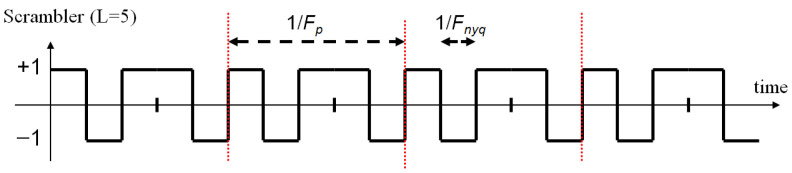
Illustration of the scrambling signal.

Figure 2 illustrates, in a very simplified case (L=5), an example of scrambling signal. It is formed from a length-*L* binary pseudo-random sequence, which is repeated. In the time domain, the duration of a binary symbol is $1/F_{nyq}$, and the period of the signal is $1/F_{p}$ (where $F_{nyq}$ and $F_{p}$ are the frequencies defined above).

**Figure 3:**
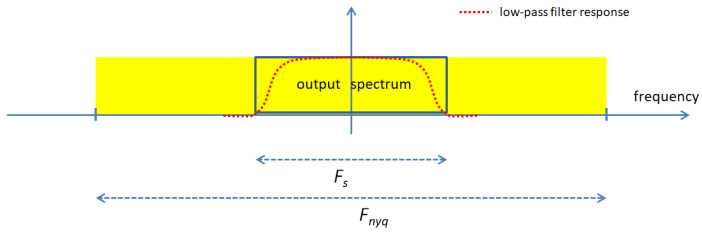
Illustration of the low-pass filter response and output spectrum.

Figure 3 illustrates the low-pass filter response. The scrambled signal, which occupies a frequency band of width $F_{nyq}$, goes through a low-pass filter. The filter output is sampled at a frequency $F_{s}$, high enough to avoid aliasing.

The scrambler and the ADC are controlled by a common central clock to avoid synchronization problems.

Reconstruction of the input signal, and calibration of the system, are based on the information provided by a block of *a* output samples. In order to avoid unnecessary mathematical complications, the value of *a* is chosen such that it corresponds to an integer number *K* of scrambling patterns, then a=KL/b. This output block then corresponds to N=KL scrambler samples (and also to *N* input samples if the input signal were sampled at $F_{nyq}$). The size of the block determines the frequency resolution $F_{s}/a=F_{nyq}/N$.

**Figure 4:**
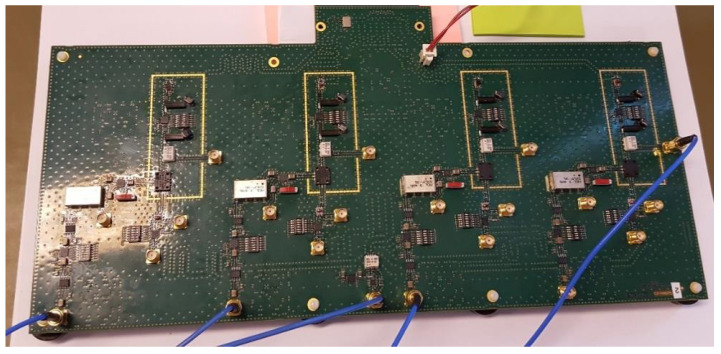
Our analog acquisition board.

For our experiments on real-world data, we designed a 4-channel MWC analog board (Figure 4), which was described in more detail in a previous paper [19]. The scramblers are sampled at $F_{nyq}=1$ GHz and their length is L=96. Therefore, their repetition frequency is $F_{p}=F_{nyq}/L=10.41667$ MHz. The device is then able to monitor a wideband spectrum of 1 GHz.

The prototype includes M=4 physical channels. Each channel features an M1-0008 mixer from MArki^©^, and an SXLP-36+ low-pass filter from Mini-Circuits^©^. The filter cut-off frequency is 40 MHz (at -3 dB). The SXLP-36+ filter was chosen because it is quite close to the ideal low-pass filter. Indeed, it has a sharp cut-off, linear phase and flat band (attenuation < 0.5 dB) in the frequency range (0–36) MHz. The ADC sampling frequency is $F_{s}=10F_{p}=104.1667MHz$ (at $F_{s}/2$ the filter attenuation is more than 30 dB); therefore, the downsampling factor is b=9.6. Table 1 sums up the main parameters.

**Figure 5:**
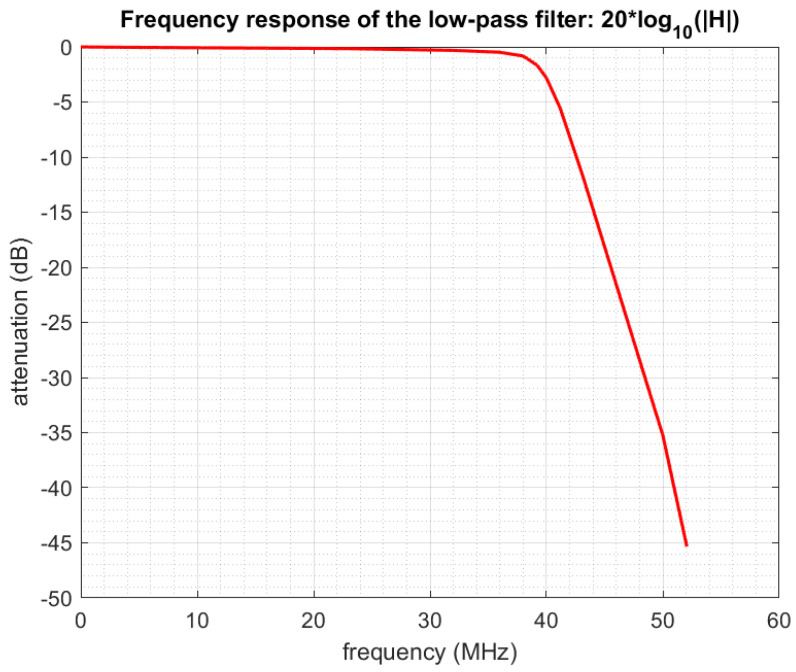
Frequency response of the low-pass filter.

**Figure 6:**
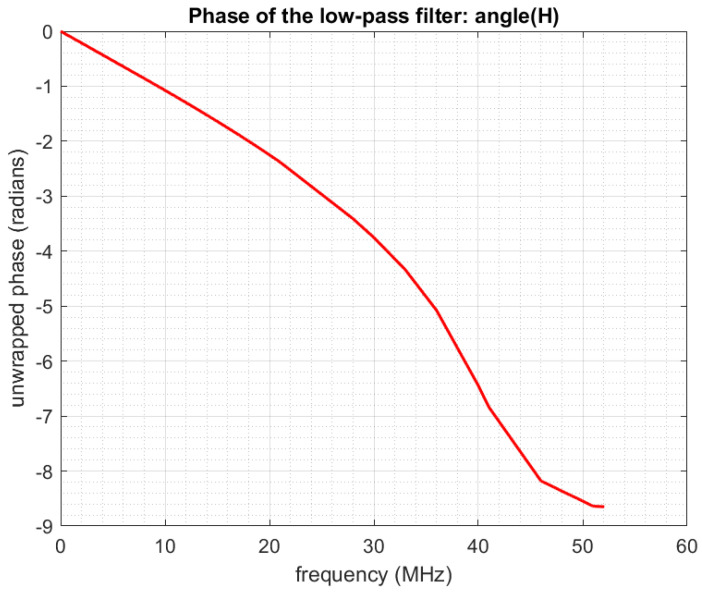
Phase of the low-pass filter.

The frequency response of the low-pass filter implemented on our acquisition board is shown in Figure 5 and its phase i n Figure 6. Details on filter calibration can be found in one of our previous papers [30].

$F_{nyq}$ being the Nyquist frequency of the input signal, we can consider a digital equivalent model at $F_{nyq}$ without loss of information. Furthermore, as previously mentioned, since calibration and signal processing are always performed on a limited amount of data in real-world applications, we can consider an input block of *N* samples (at $F_{nyq}$).

Modern implementations of the FFT [31] contain a special code to handle splittings not only of size 2 but also of sizes 3 (and sometimes 5 and 7). Therefore, for the efficiency of the FFT, we will preferably choose block sizes whose prime factors belong to {2,3,5,7}. In our experiments, we have taken K=448, $N=KL=43,008=2^{11}\times 3\times 7$ and $a=4480=2^{9}\times 5\times 7$. The frequency resolution is then $F_{nyq}/N≃23$ kHz, which is far sufficient unless we would like to detect extreme narrow-band transmitters.

## 4. System Model and Matrix Representation

### 4.1. System Equations in the Time Domain

Let us note:

- *x* the vector representing the input signal.
- *s* the vector representing the scrambling signal.
- *v* the vector representing the scrambler output.
- *h* the vector representing the low-pass filter impulse response.
- *w* the vector representing the low-pass filter output.
- *y* the 1×a vector containing the digital output samples (at $F_{s}$).

**Figure 7:**
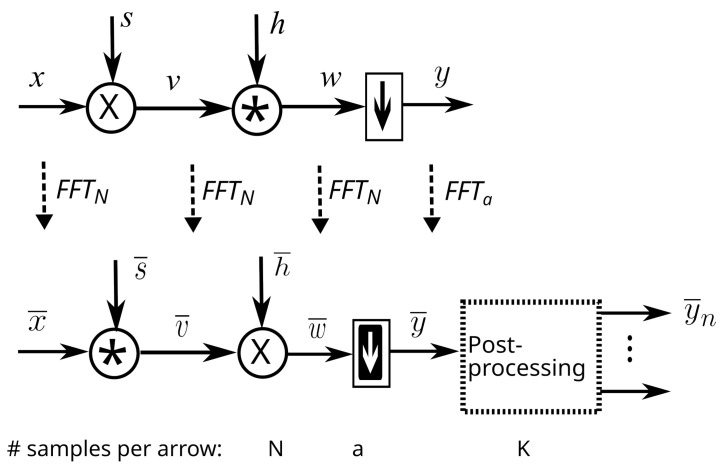
Principle of the system, using vector notations, in the time and frequency domains.

All of these vectors, except for *y*, are (1×N) vectors and represent the signals at $F_{nyq}$ samples per second. In Figure 7, we show the links between these vectors. In the time domain (top of the figure), the signals are represented by vectors. Symbol * stands for cyclic convolution. These vectors can be transposed in the frequency domain using a multiplication by matrix $F_{N}$ or $F_{a}$. A post-processing, described later, is then performed in the frequency domain. The post-processing outputs *q* vectors ${\tilde{y}}_{n}$ of size 1×K.

In the figure, we have used different symbols for down-sampling because the operation in the time and frequency domains are different. For instance, when *b* is an integer, down-sampling in the time domain consists of picking one sample out of *b* while its equivalent in the frequency domain is a multiplication of the down-sampling matrix Ξ, which will be defined later.

Notations used below have already been defined in Section 2. Since the system is linear, in the time domain, we have

(39) y=xB

where *B* is an N×a matrix. The structure of *B* can be easily computed from the system model (Figure 7):

(40) $$B=D_{s}C_{h}\left(I_{a}⊗1_{b}^{T}\right)$$

Indeed, the scrambler output is given by:

(41) $$v=xD_{s}$$

The filter output is:

(42) $$w=vC_{h}$$

For the moment, let us consider that *b* is an integer (we will see later that this is not a requirement). In that case, down-sampling consists of picking one sample out of *b* in *w*. Mathematically, that is:

(43) $$y=w\left(I_{a}⊗1_{b}^{T}\right)$$

Otherwise, down-sampling can be modeled using an interpolation matrix. However, we will not detail this because only the equations in the frequency domain will be useful for our purpose. We will see later that in the frequency domain, thanks to the presence of a low pass filter, *b* being an integer is not a requirement anymore.

### 4.2. System Equations in the Frequency Domain

Multiplying Equation (39) by $F_{a}$ and inserting the identity $F_{N}F_{N}^{-1}$ where appropriate, we obtain:

(44) $$yF_{a}=\left(xF_{N}\right)\left(F_{N}^{-1}BF_{a}\right)$$

That is

(45) $$\bar{y}=\bar{x}A$$

where *A* is an N×a matrix.

(46) $$A=F_{N}^{-1}BF_{a}$$

The structure of *A* can be detailed further. Replacing *B* with its expression (Equation (40)) and inserting the identity $F_{N}F_{N}^{-1}$ where it is appropriate, we obtain:

(47) $$\begin{array}{cc} A & =\left(F_{N}^{-1}D_{s}F_{N}\right)\left(F_{N}^{-1}C_{h}F_{N}\right)\left(F_{N}^{-1}\left(I_{a}⊗1_{b}^{T}\right)F_{a}\right) \end{array}$$

Then, using Equations (15) and (16) we obtain:

(48) $$A=\frac{1}{N}C_{\bar{s}}D_{\bar{h}}\Xi$$

As proved in the appendix (see Appendix A.1), the frequency-domain down-sampling matrix Ξ is:

(49) $$\Xi =\frac{1}{b}\theta_{b}^{T}⊗I_{a}$$

That is:

(50) $$\Xi =\frac{1}{b}\left(\begin{array}{c} I_{a}\\ \vdots \\ I_{a} \end{array}\right)$$

where sub-matrix $I_{a}$ is repeated *b* times. Here we remind that *h* is a low-pass filter. Since the ADC sampling frequency is $F_{s}$, we assume that the elements of $\bar{h}$, which correspond to frequencies outside the interval $]-F_{s}/2,F_{s}/2$ [are almost null. Since $\bar{h}$ contains *N* elements, the frequency resolution is $F_{nyq}/N$, so $F_{s}/2$ corresponds to index $\left(F_{s}/2\right)/\left(F_{nyq}/N\right)=N/\left(2b\right)$, that is, a/2. Let us note

(51) $$c=\left\lfloor a/2\right\rfloor$$

and

(52) δ=amod2

Therefore, the elements of $\bar{h}$ are almost null for indices outside the interval [−c,c+δ] (the indices are considered modulo *N*). Hence, we can redefine Ξ as the N×a matrix below:

(53) $$\Xi =\frac{1}{b}\left(\begin{array}{cc} I_{c+\delta } & 0\\ 0 & 0\\ 0 & I_{c} \end{array}\right)$$

without changing the product $D_{\bar{h}}\Xi$. Here, the zeros stand for null sub-matrices. We see that, thanks to the low-pass pass filter which leads to this structure of Ξ, it is not required any more that *b* is an integer (this requirement was only due to the need for an integer number of occurrences of $I_{a}$ in Equation (50)).

Finally, let us define the (1×a) vector $\tilde{h}$:

(54) $$\tilde{h}=\left[\begin{array}{cccccc} {\bar{h}}_{0} & \cdots & {\bar{h}}_{c+\delta -1} & {\bar{h}}_{-c} & \cdots & {\bar{h}}_{-1} \end{array}\right]$$

where the indices are modulo *N*. We then have:

(55) $$D_{\bar{h}}\Xi =\Xi D_{\tilde{h}}$$

and the expression of matrix *A* becomes:

(56) $$A=\frac{1}{N}C_{\bar{s}}\Xi D_{\tilde{h}}$$

### 4.3. Unconstrained System Equations in the Time Domain

Now we can go back to the time domain to obtain a matrix *B*, which does not require *b* being an integer. We have:

(57) $$\begin{array}{cc} y & =\bar{y}F_{a}^{-1} \end{array}$$

(58) $$\begin{array}{cc} \; & =\bar{x}AF_{a}^{-1} \end{array}$$

(59) $$\begin{array}{cc} \; & =\left(\bar{x}F_{N}^{-1}\right)\left(F_{N}AF_{a}^{-1}\right) \end{array}$$

(60) $$\begin{array}{cc} \; & =xB \end{array}$$

where

(61) $$B=F_{N}AF_{a}^{-1}$$

### 4.4. Fast Simulation of the Acquisition System

**Figure 8:**
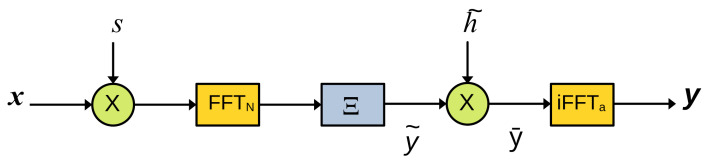
Fast simulation. Dotted arrows are for *a* elements, full arrows are for *N* elements.

A first interest of the linear algebra model is that it makes the design of a fast simulator obvious. Indeed, multiplication by a diagonal matrix *D* is efficiently implemented as an element-by-element vectors product, and multiplication by a Fourier matrix *F* (or its inverse) is efficiently implemented by Fast Fourier Transform (FFT). On the contrary, multiplications by circulant matrices *C* should be avoided because of their computational cost. Then, the method to design a fast simulator is to insert identities $FF^{-1}$ or $F^{-1}F$ where it is appropriate in order to suppress the circulant matrices. For instance, we have:

(62) $$\begin{array}{cc} y & =xB \end{array}$$

(63) $$\begin{array}{cc} \; & =xF_{N}\frac{1}{N}C_{\bar{s}}\Xi D_{\tilde{h}}F_{a}^{-1} \end{array}$$

(64) $$\begin{array}{cc} \; & =x\left(F_{N}\frac{1}{N}C_{\bar{s}}F_{N}^{-1}\right)F_{N}\Xi D_{\tilde{h}}F_{a}^{-1} \end{array}$$

(65) $$\begin{array}{cc} \; & =xD_{s}F_{N}\Xi D_{\tilde{h}}F_{a}^{-1} \end{array}$$

using Equation (16). Here we have only fast operations, as shown in Figure 8.

## 5. Equivalent Model and Post-Processing

### 5.1. Equivalent Model

Until now, we have not taken advantage of the periodicity of the scrambler. This opens the way to an equivalent model with interesting properties.

The scrambler is a (1×N) vector *s*, which contains *K* replica of a basic waveform represented by a (1×L) vector *p*. Then, the scrambler can be written:

(66) $$s=\theta_{K}⊗p$$

and we have (see proof in Appendix A.2)

(67) $$\bar{s}=K\bar{p}⊗1_{K}$$

It follows that $\bar{s}$ is sparse (only one element out of *K* is non-zero). It will be easier to take benefit of the sparsity of $\bar{s}$ if we permute $\bar{x}$ and $\bar{s}$ in the expression of $\bar{y}$:

(68) $$\begin{array}{cc} \bar{y} & =\frac{1}{N}\bar{x}C_{\bar{s}}\Xi D_{\tilde{h}} \end{array}$$

(69) $$\begin{array}{cc} \; & =\frac{1}{N}\bar{s}C_{\bar{x}}\Xi D_{\tilde{h}} \end{array}$$

The proof is trivial: since the multiplication is commutative, we can permute *x* and *s* (see Figure 7); therefore, we can also permute $\bar{x}$ and $\bar{s}$.

Let us define the L×N matrix $C_{\bar{x}}^{\left(K\right)}$ obtained by picking one row out of *K* in $C_{\bar{x}}$. That is:

(70) $$C_{\bar{x}}^{\left(K\right)}=\left(I_{L}⊗1_{K}\right)C_{\bar{x}}$$

More explicitly, that is:

(71) $$C_{\bar{x}}^{\left(K\right)}=\left(\begin{array}{ccc} {\bar{x}}_{0} & \cdots & {\bar{x}}_{N-1}\\ {\bar{x}}_{-K} & \cdots & {\bar{x}}_{N-K-1}\\ \vdots & \; & \vdots \\ {\bar{x}}_{-(L-1)K} & \cdots & {\bar{x}}_{K-1} \end{array}\right)$$

where the indices are considered modulo *N*.

Let us denote

(72) $$\tilde{p}=\frac{1}{L}\bar{p}$$

Using Equation (67), the mixer output becomes:

(73) $$\begin{array}{cc} \frac{1}{N}\bar{s}C_{\bar{x}} & =\frac{1}{N}\left(K\bar{p}⊗1_{K}\right)C_{\bar{x}} \end{array}$$

(74) $$\begin{array}{cc} \; & =\frac{1}{L}\left(\left(\bar{p}I_{L}\right)⊗1_{K}\right)C_{\bar{x}} \end{array}$$

(75) $$\begin{array}{cc} \; & =\frac{1}{L}\bar{p}\left(I_{L}⊗1_{K}\right)C_{\bar{x}} \end{array}$$

(76) $$\begin{array}{cc} \; & =\tilde{p}C_{\bar{x}}^{\left(K\right)} \end{array}$$

An interesting property of matrix $C_{\bar{x}}^{\left(K\right)}$, which will be exploited later, is (see proof in Appendix A.3):

(77) $$C_{\bar{x}}^{\left(K\right)}J_{N}^{K}=J_{L}C_{\bar{x}}^{\left(K\right)}$$

Finally, let us define

(78) $$\tilde{y}=\bar{y}D_{1/\tilde{h}}$$

We then have:

(79) $$\begin{array}{cc} \tilde{y} & =\frac{1}{N}\bar{s}C_{\bar{x}}\Xi \end{array}$$

(80) $$\begin{array}{cc} \; & =\tilde{p}C_{\bar{x}}^{\left(K\right)}\Xi \end{array}$$

### 5.2. Post-Processing

The post-processing extracts frequency blocks of *K* samples from $\tilde{y}$.

Using definition (35), let us note $S_{n}$ the a×K selection matrix below

(81) $$S_{n}=bS_{a,K}^{\left(r+nK\right)}$$

and $R_{n}$ the N×K selection matrix below

(82) $$R_{n}=S_{N,K}^{\left(r+nK\right)}$$

Denoting ${\tilde{y}}_{n}$ a 1×K vector representing the selected data, we have:

(83) $${\tilde{y}}_{n}=\tilde{y}S_{n}$$

${\tilde{y}}_{n}$ contains the elements of $\tilde{y}$ whose indices (modulo *a*) are in the interval Φ=[r+nK,r+nK+K−1].

The indices are considered modulo *a*, so *r* may be negative. We will consider that Φ⊂[−c,c+δ], so

(84) $$\begin{array}{cc} \Xi S_{n} & =\Xi bS_{a,K}^{\left(r+nK\right)} \end{array}$$

(85) $$\begin{array}{cc} \; & =S_{N,K}^{\left(r+nK\right)} \end{array}$$

(86) $$\begin{array}{cc} \; & =R_{n} \end{array}$$

We can note that:

(87) $$R_{n}=J_{N}^{-nK}R_{0}$$

This is a matrix similar to $R_{0}$ but with sub-matrix $I_{K}$ circularly shifted nK positions downwards. We can note that we have also:

(88) $$R_{n+1}=J_{N}^{-K}R_{n}$$

Eventually, using Equations (73) and (77) we have:

(89) $$\begin{array}{cc} {\tilde{y}}_{n} & =\tilde{p}C_{\bar{x}}^{\left(K\right)}\Xi S_{n} \end{array}$$

(90) $$\begin{array}{cc} \; & =\tilde{p}C_{\bar{x}}^{\left(K\right)}R_{n} \end{array}$$

(91) $$\begin{array}{cc} \; & =\tilde{p}C_{\bar{x}}^{\left(K\right)}J_{N}^{-nK}R_{0} \end{array}$$

(92) $$\begin{array}{cc} \; & =\left(\tilde{p}J_{L}^{-n}\right)\left(C_{\bar{x}}^{\left(K\right)}R_{0}\right) \end{array}$$

(93) $$\begin{array}{cc} \; & ={\tilde{p}}_{n}Z_{\bar{x}} \end{array}$$

where

(94) $${\tilde{p}}_{n}=\tilde{p}J_{L}^{-n}$$

and

(95) $$Z_{\bar{x}}=C_{\bar{x}}^{\left(K\right)}R_{0}$$

More explicitly, $Z_{\bar{x}}$ is the L×K matrix below:

(96) $$Z_{\bar{x}}=\left(\begin{array}{ccc} {\bar{x}}_{r} & \cdots & {\bar{x}}_{r+K-1}\\ {\bar{x}}_{r-K} & \cdots & {\bar{x}}_{r-1}\\ \vdots & \; & \vdots \\ {\bar{x}}_{r-(L-1)K} & \cdots & {\bar{x}}_{r-(L-2)K-1} \end{array}\right)$$

were the indices are considered modulo *N*. The interesting feature in Equation (89) is that $Z_{\bar{x}}$ does not depend on *n*. Hence, we can take *q* different values of *n* and write

(97) $$\left(\begin{array}{c} \vdots \\ {\tilde{y}}_{n}\\ \vdots \end{array}\right)=\left(\begin{array}{c} \vdots \\ {\tilde{p}}_{n}\\ \vdots \end{array}\right)Z_{\bar{x}}$$

assuming we know the filter frequency response (which should be the case because the filter is part of the acquisition system). More compactly, this fundamental equation can be noted:

(98) Y=PZ

were *Y* is the (q×K) matrix below:

(99) $$Y=\left(\begin{array}{c} \vdots \\ {\tilde{y}}_{n}\\ \vdots \end{array}\right)$$

*P* is the (q×L) matrix below:

(100) $$P=\left(\begin{array}{c} \vdots \\ {\tilde{p}}_{n}\\ \vdots \end{array}\right)$$

and *Z* is the (L×K) matrix below:

(101) $$Z=Z_{\bar{x}}$$

Therefore, the sizes of the matrices appearing in Equation (98) are (q×K),(q×L),(L×K). Then, if the number of non-zero rows in *Z* is less than *q* the matrix *Z* can be reconstructed from this equation using an algorithm such as OMP [29]. Eventually, from *Z* we can retrieve $\bar{x}$ as shown below. Indeed, it is easy to see that $\bar{x}$ can be rebuilt from *Z* with

(102) $$\bar{x}=vec\left(A_{L}Z\right)J_{N}^{r+K}$$

where $A_{L}$ is the K×K anti-diagonal matrix:

(103) $$A_{L}=\left(\begin{array}{cccc} 0 & \cdots & 0 & 1\\ \vdots & & 1 & 0\\ 0 & & & \vdots \\ 1 & 0 & \cdots & 0 \end{array}\right)$$

The effect of the multiplication of a matrix by $A_{L}$ on the left is to reverse the order of its rows.

If we have *M* channels instead of one in the physical system, the number of rows of *Y* becomes qM; hence, we can theoretically rebuild the signal if the number of non-zero rows in *Z* is less than qM.

Let us note q=2ρ+τ the Euclidean division of *q* by 2. In our experiments, we have set r=0 for *q* even and $r=-\left\lfloor K/2\right\rfloor$ for *q* odd. For *n* we take the integers in the interval −ρ to ρ+τ−1. This choice, while not compulsory, is designed to take into account equally distributed values around frequency 0 in $\tilde{y}$, which is a priori the best choice. Indeed, the indices of the samples taken into account go from r−ρK to r+(ρ+τ)K−1, that is:

- For *q* odd: from $-\rho K-\left\lfloor K/2\right\rfloor$ to $\rho K+\left\lceil K/2\right\rceil -1$
- For *q* even: from −ρK to ρK−1

**Figure 9:**
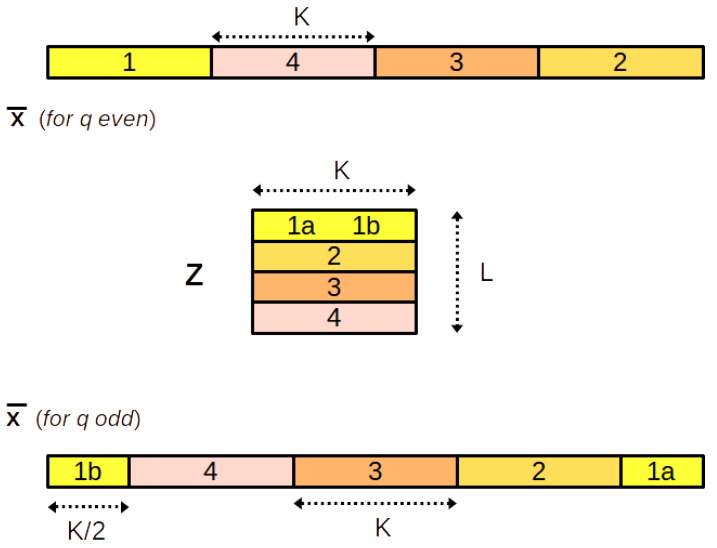
Arrangement of the elements of $\bar{x}$ into matrix $Z_{\bar{x}}$, for L=4.

**Figure 10:**
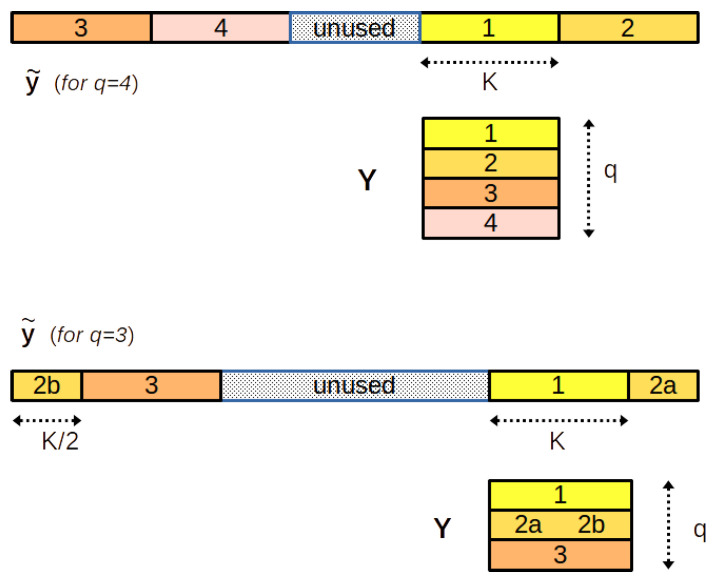
Arrangement of the elements of $\tilde{y}$ into matrix Y, for q=4 and q=3.

For this choice, Figure 9 illustrates how the elements of $\bar{x}$ are arranged into matrix $Z_{\bar{x}}$ and Figure 10 illustrates how the elements of $\tilde{y}$ are arranged into matrix *Y*.

### 5.3. Application of the Equivalent Model to Reconstruction

The input of the reconstruction algorithm is the vector *y* provided by the acquisition device. The output is an estimation of $\bar{x}$.

We assume that:

- The frequency response $\bar{h}$ of the low-pass filter is known (or has been estimated). Then $\tilde{h}$ can be precomputed using Equation (54).
- Matrix *P* has been precomputed, using Equations (72), (94) and (100), or (better) has been estimated by the calibration process (see Section 6).

The procedure is as follows:

1. Using an *a*-points FFT compute $\bar{y}$
2. Compensate the filter by computing $\tilde{y}$ (Equation (78))
3. Extract *q* sub-vectors ${\tilde{y}}_{n}$ from $\tilde{y}$ (Equation (83))
4. Compute matrix *Y* (Equation (99))
5. Use a compressed sensing algorithm, such as OMP [29], to estimate matrix *Z* from Equation (98)
6. Reconstruct $\bar{x}$ from *Z* using Equation (102)

As an illustration of how the linear algebra model makes things simple from the programming point of view, this is the Octave program, which builds matrix *Y* from y and then obtains *x* from the reconstructed matrix *Z*:

ytilde = fft(y,[],2)./htilde;

ind = 1+mod(r+[-rho*K:(rho+tau)*K-1], a);

Y = reshape(ytilde(ind),K,q).’;

% Insert here estimation of Z from Y and P (using OMP, for instance)

xbs = reshape(Z(L:-1:1,:).’,1,N);

xb = circshift(xbs,[0 r+K]);

x = ifft(xb,[],2);

If there are M>1 physical channels, the q×K matrices *Y* corresponding to each channel are stacked vertically, leading to a qM×K matrix *Y*.

### 5.4. Interest of *Q* Even

The linear algebra model allows even values of *q*, instead of previous models that required *q* to be odd. The main interest is that it puts lower constraints on the design of the acquisition board. If the acquisition board is already available, it may also allow a better use of the MWC output data if the acquisition board is not perfectly optimized.

Let us consider our own acquisition board, which was designed before we established the linear-algebra model. We remind that the ADC sampling frequency is $F_{s}≃104.2$ MHz and the scramblers repetition frequency is $F_{p}=F_{nyq}/L≃10.42$ MHz. Using $F_{s}=F_{nyq}/b$ and N=KL=ab, it is easy to see that $F_{p}=KF_{s}/a$. In the frequency domain, the *a* output samples correspond to $F_{s}$, then qK output samples correspond to $qKF_{s}/a=qF_{p}$.

- With q=7, we put into *Y* a total of qK samples corresponding to a frequency half-band $qF_{p}/2=36.47$ MHz. This choice perfectly fits the frequency response of the low-pass filter, which is an almost perfectly flat and linear phase in [DC-36MHz] (see Figure 5 and Figure 6).
- With q=6, we would put into *Y* samples corresponding to a frequency half-band $qF_{p}/2=31.26$ MHz. This corresponds to an even better area of the filter response, but in doing this, we would not use all the available information.
- On the contrary, with q=8, we would put it into *Y* samples corresponding to a frequency half-band $qF_{p}/2=41.68$ MHz. This allows us to take more information into account, but we see that we take into account some samples corresponding to a lower quality of the filter response.

This result is not surprising, because our acquisition board was designed and optimized for q=7, but for a future design of a new board, the possibility to have *q* may even be interesting because it puts fewer constraints on the choice of the commercial filters.

Indeed, applying the proposed method to our analog board did not need any change because odd values of *q* are allowed by our method. If a new analog board were to be designed, our method adds an additional degree of freedom because it allows even values of *q* also. This would allow, for example, the designer of the analog board to use a low-pass filter with a cut-off frequency of about 34 MHz (using q=6) instead of 40 MHz (using q=7).

## 6. Application to Fast Calibration

### 6.1. Proposed Approach

The objective of calibration is to estimate the true matrix *P*. Indeed, for real-world applications, using the theoretical matrix (Equation (100)) leads to very poor results [13]. In a previous paper [19], we proposed an approach that uses a single wideband signal for calibration, contrary to previous approaches that required successive injections of sinusoids in the system. In that paper, we presented spectrum reconstruction performances and examples of spectrum reconstruction obtained with our calibration method. In the present paper, we will mainly focus on simplifying and speeding up the method thanks to the linear algebra model.

The signal we used for calibration has a spectrum that is totally flat in the

$\left[-F_{nyq}/2,F_{nyq/2}\right]$ frequency band and random phase. Compared to methods based on iterative injections of sinusoidal waves, our new calibration method is time-saving and is more practical in terms of the simplicity of implementation because it requires a single measurement to perform the calibration. The calibration method uses advanced resynchronization preprocessing. Our calibration method offers slightly better spectrum reconstruction performances compared to reference method [13].

If we know matrices *Y* and *Z*, using Equation (98), we can estimate matrix *P* by:

(104) $$\hat{P}=YZ^{+}$$

where $Z^{+}$ is the Moore–Penrose pseudo-inverse [27] of *Z*. Matrix *Y* depends on the MWC outputs, and matrix *Z* depends on its input signal. The problem in a real-world context is that we cannot reliably synchronize the input of the MWC with the ADC sampling, which provides the output, and even if a costly synchronization device was implemented, there are delays in the analog board itself, which are intractable. Then, the input signal must be designed such that a synchronization can be performed numerically. Otherwise, in Equation (104), we would multiply matrices *Y* and $Z^{+}$ corresponding to desynchronized data, which would make no sense.

In order to allow an efficient numerical synchronization, we used, for the MWC input, a periodical signal with a flat spectrum and random phase. More precisely, the period of this signal corresponds to the chosen block size, that is $N/F_{nyq}$, and one period can be represented by a length-*N* row vector *x*. This vector is generated as follows:

1. A length-*N* vector $\bar{x}$ is generated such that, for any of its elements $\bar{x}\left(k\right)$, we have $\left|\bar{x}\left(k\right)\right|=1$ and $Arg\left(\bar{x}\left(k\right)\right)$ is random in [0,2π] under the constraint $Arg\left(\bar{x}\left(-k\right)\right)=-Arg\left(\bar{x}\left(k\right)\right)$ (this constraint ensures that *x* is real).
2. *x* is deduced from $\bar{x}$ by an inverse FFT: $x=\bar{x}F_{N}^{-1}$

Reminding that matrix *Z* contains the elements of $\bar{x}$ (Equation (101)), the constant modulus $\left|\bar{x}\left(k\right)\right|=1$ ensures that no element of *Z* is privileged or disadvantaged by the input signal. Furthermore, the random phase ensures that the input signal has good localization properties, which is desired for efficient synchronization. Finally, choosing a periodic signal has a strong advantage: a time shift of a block taken on the input signal is equivalent to a cyclic permutation of vector *x*.

On the programming point of view, building *Z* from *x* is very simple:

xb = fft(x, [], 2);

xbs = circshift(xb,[0 -(r+K)]);

Z(L:-1:1,:) = reshape(xbs,K,L).’;

In the following, we will note $x_{0}=x$ as the signal pattern and $x_{d}$ a cyclic permutation, *d* positions to the right, of the pattern. This means that

(105) $$x_{d}=xJ_{N}^{d}$$

The procedure that we propose is as follows:

1. Feed the acquisition device with a periodic signal, which is a repetition of a known pattern $x_{0}$
2. Record *a* samples at the output of the acquisition device (this is vector *y*), then compute matrix *Y* using steps 1 to 4 of the reconstruction procedure.
3. Perform cyclic permutations of the input pattern $x_{0}$. For each vector $x_{d}$, compute matrix $Z_{d}=Z_{{\bar{x}}_{d}}$ (using Equations (96) and (101)) and then $\hat{P}$ (using Equation (104)).

Let us note the residue

(106) $$R_{d}=Y-{\hat{P}}_{d}Z_{d}.$$

The criterion to determine the best cyclic permutation of the input pattern $x_{0}$ is the inverse Frobenius norm of $R_{d}$ (the Frobenius norm is the square root of the sum of the square modules of the elements of the matrix).

The computational cost per iteration (i.e., per value of *d* tested) can be estimated as follows:

- A FFT is required to compute ${\bar{x}}_{d}$, that is $LK\log_{2}\left(LK\right)$ flops (because N=LK).
- Computation of the pseudo-inverse $Z_{d}^{+}$: $7KL^{2}+20L^{3}$ flops (see Section 2.7).
- Computation of $\hat{P}=YZ_{d}^{+}$ (the sizes of the matrices are qM×K and K×L): qMKL flops.
- Computation of $\hat{P}Z_{d}$ requires qMLK flops.
- Computation of the Frobenius norm requires KL flops

Globally, since the computation of the Frobenius norm can be neglected compared to the other terms, the algorithm requires about $L(2qMK+7LK+20L^{2}+K\log_{2}\left(LK\right))$ flops per iteration.

### 6.2. Fast Update of Matrix Z

While evaluating all possible shifts *d*, computation of matrix $Z_{d}=Z_{{\bar{x}}_{d}}$ requires an *N*-points FFT to obtain ${\bar{x}}_{d}$, which requires approximately $Nlog_{2}\left(N\right)$ multiplication at each iteration. However, we can reduce the complexity just by computing the first matrix and then updating it at each iteration as described below. Let us consider a vector $x_{d}$, which is a cyclic permutation, *d* positions to the right of pattern *x*. We have:

(107) $$x_{d}=x_{0}J_{N}^{d}$$

Then

(108) $$\begin{array}{cc} {\bar{x}}_{d} & =x_{0}J_{N}^{d}F_{N} \end{array}$$

(109) $$\begin{array}{cc} \; & =x_{0}F_{N}F_{N}^{-1}J_{N}^{d}F_{N} \end{array}$$

(110) $$\begin{array}{cc} \; & ={\bar{x}}_{0}D_{{\bar{\alpha }}_{d}} \end{array}$$

where

(111) $$\alpha_{d}=\left[\underset{d}{\underset{︸}{0\cdots 0}}10\cdots 0\right]$$

Indeed, since $J_{N}^{d}=C_{\alpha_{d}}$, using Equation (15) we have:

(112) $$\begin{array}{cc} F_{N}^{-1}J_{N}^{d}F_{N} & =F_{N}^{-1}C_{\alpha_{d}}F_{N} \end{array}$$

(113) $$\begin{array}{cc} \; & =D_{{\bar{\alpha }}_{d}} \end{array}$$

Since ${\bar{\alpha }}_{d}=\alpha_{d}F_{N}$, it is easy to see that ${\bar{\alpha }}_{d}$ is the ${\left(d+1\right)}^{th}$ row of $F_{N}$, that is (see Equations (10) and (11)):

(114) $${\bar{\alpha }}_{d}=\left[\begin{array}{ccccc} 1 & \omega^{d} & \omega^{2d} & \cdots & \omega^{(N-1)d} \end{array}\right]$$

Then, we have:

(115) $$\begin{array}{cc} Z_{d} & =Z_{{\bar{\alpha }}_{d}•{\bar{x}}_{0}} \end{array}$$

(116) $$\begin{array}{cc} \; & =Z_{{\bar{\alpha }}_{d}}•Z_{0} \end{array}$$

where • stands for element by element multiplication. This equation can also be written

(117) $$Z_{d}=\omega^{-rd}\Theta_{d}^{*}Z_{0}\Omega_{d}$$

with

(118) $$\Theta_{d}=diag\left[\begin{array}{ccccc} 1 & \omega^{Kd} & \omega^{2Kd} & \cdots & \omega^{(L-1)Kd} \end{array}\right]$$

(119) $$\Omega_{d}=diag\left[\begin{array}{ccccc} 1 & \omega^{d} & \omega^{2d} & \cdots & \omega^{(K-1)d} \end{array}\right]$$

To see where this formula comes from, we must remind that multiplication by a diagonal matrix on the left (right) multiplies the rows (columns) by the elements of the diagonal. Then, denoting $\omega_{d}=\omega^{d}$ we can see that:

(120) $$\begin{array}{cc} \omega_{d}^{-r}vec{\left(\Theta_{d}\right)}^{*}vec\left(\Omega_{d}\right) & =\omega_{d}^{-r}\left[\begin{array}{c} 1\\ \omega_{d}^{-K}\\ \cdots \\ \omega_{d}^{-(L-1)K} \end{array}\right]\left[\begin{array}{cccc} 1 & \omega_{d} & \cdots & \omega_{d}^{K} \end{array}\right] \end{array}$$

(121) $$\begin{array}{cc} \; & =\omega_{d}^{-r}\left[\begin{array}{cccc} 1 & \omega_{d} & \cdots & \omega_{d}^{K-1}\\ \omega_{d}^{-K} & \omega_{d}^{-K+1} & \cdots & \omega_{d}^{-1}\\ \vdots & \vdots & \; & \vdots \\ \omega_{d}^{-(L-1)K} & \omega_{d}^{-(L-1)K+1} & \cdots & \omega_{d}^{-(L-2)K-1} \end{array}\right] \end{array}$$

(122) $$\begin{array}{cc} \; & =Z_{{\bar{\alpha }}_{d}} \end{array}$$

If we evaluate by step *g*, we can use:

(123) $$Z_{d}=Z_{{\bar{\alpha }}_{g}}•Z_{d-g}$$

Matrix $Z_{{\bar{\alpha }}_{g}}$ can be precomputed. Therefore, at each iteration, we need only *N* multiplications, which is less complex than computing ${\bar{x}}_{d}$ each time. This update requires only N=LK multiplications at each iteration, instead of approximately $Nlog_{2}\left(N\right)$.

If we want to allow sub-sample precision (i.e., g<1), we just have to note η=N/2 and write ${\bar{\alpha }}_{g}$ as follows:

(124) $${\bar{\alpha }}_{g}=\left[\begin{array}{ccccccc} 1 & \omega^{g} & \cdots & \omega^{\eta -1} & \omega^{-\eta g} & \cdots & \omega^{-g} \end{array}\right]$$

This is very interesting because sub-sample precision is then allowed without any additional cost due to oversampling (with this method, no oversampling is required). Matrix $Z_{{\bar{\alpha }}_{g}}$ is computed as follows:

alpha = exp(-i*2*pi*g*[0:eta-1 -eta:-1]/N);

alpha = circshift(alpha,[0 -(r+K)]);

Zalpha(L:-1:1,:) = reshape(alpha,K,L).’;

Then, at each iteration, updating matrix *Z* is achieved by:

Z = Z .* Zalpha;

### 6.3. Fast Update of Matrix $Z^{+}$

The approach is similar to the pseudo-inverse: we can reduce the complexity just by computing the first pseudo-inverse matrix and then updating it at each iteration, as described below. This update requires only *N* multiplications at each iteration.

According to discussions above, denoting $Z_{0}=USV^{*}$ as the SVD of $Z_{0}$, we have

(125) $$\begin{array}{cc} Z_{d} & =\omega^{-rd}\Theta_{d}^{*}\left(USV^{*}\right)\Omega_{d} \end{array}$$

(126) $$\begin{array}{cc} \; & =\left(\omega^{-rd}\Theta_{d}^{*}U\right)S{\left(\Omega_{d}^{*}V\right)}^{*} \end{array}$$

This equation directly provides the SVD of $Z_{d}$. It follows that $Z_{d}^{+}$ is

(127) $$\begin{array}{cc} Z_{d}^{+} & =\left(\Omega_{d}^{*}V\right)S^{+}{\left(\omega^{-rd}\Theta_{d}^{*}U\right)}^{*} \end{array}$$

(128) $$\begin{array}{cc} \; & =\omega^{rd}\Omega_{d}^{*}Z_{0}^{+}\Theta_{d} \end{array}$$

This update can be realized by element-by-element multiplication:

(129) $$Z_{d}^{+}=Z_{{\bar{\alpha }}_{d}}^{*}•Z_{0}^{+}$$

If we evaluate by step *g* we can update the matrix at each iteration by:

(130) $$Z_{d}^{+}=Z_{{\bar{\alpha }}_{g}}^{*}•Z_{d-g}^{+}$$

where $Z_{{\bar{\alpha }}_{g}}^{*}$ can be precomputed.

### 6.4. Fast Synchronization

To obtain a synchronization, we must evaluate the Frobenius norm of $R_{d}$ at each iteration.

We can evaluate the computational complexity of this fast algorithm as follows:

- Updating matrix $Z_{d}$ and $Z_{d}^{+}$ requires 2LK flops.
- Computing $Y_{d}Z_{0}^{+}$ requires qMKL flops.
- Multiplying $Y_{d}Z_{0}^{+}$ by $Z_{0}$ requires qMKL flops.
- Computing the Frobenius norm requires KL flops.

Globally, the algorithm requires 2qMKL+3KL≃2qMKL flops per iteration, which is to be compared to $L(2qMK+7LK+20L^{2}+K\log_{2}\left(LK\right))$ for the previous version. The gain is, approximately:

(131) $$\begin{array}{cc} G & ≃1+\frac{7L}{2qM}+\frac{10L^{2}}{qMK}+\frac{\log_{2}\left(L\right)}{2qM}+\frac{\log_{2}\left(K\right)}{2qM} \end{array}$$

(132) $$\begin{array}{cc} \; & ≃21 \end{array}$$

Computation time on Octave is 54 s for the slow version and 1.6 s for the fast version, which is then 34 times faster.

**Figure 11:**
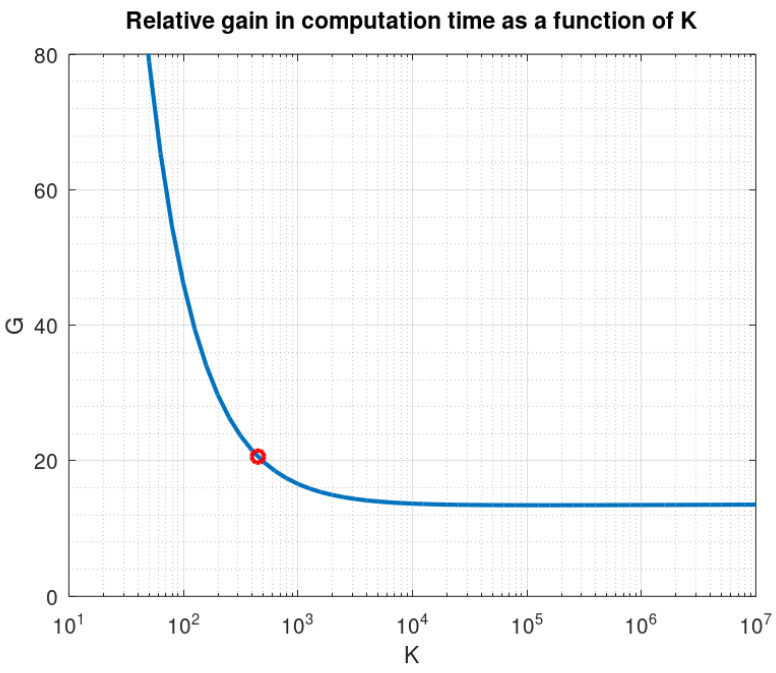
Gain as a function of *K* (the red dot shows the values corresponding to our acquisition board).

As a function of *K*, the gain decreases until $K=20\ln\left(2\right)L^{2}=127761$ (which is a huge value, not expected for real-world acquisition devices), where it reaches a minimum of 13.4, then increases slowly, behaving asymptotically as $\log_{2}\left(K\right)/\left(2qM\right)$, as shown in Figure 11.

## 7. Experimental Results

**Figure 12:**
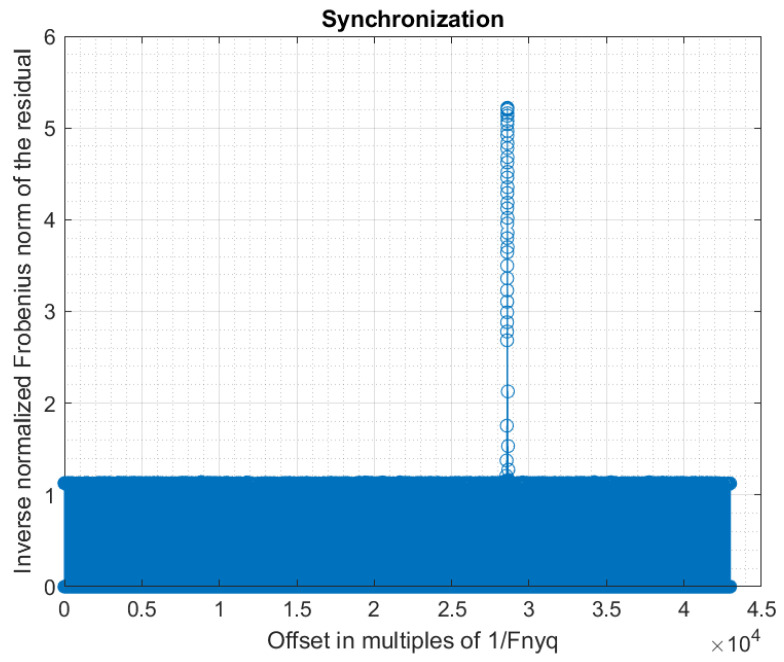
Synchronization (overview).

**Figure 13:**
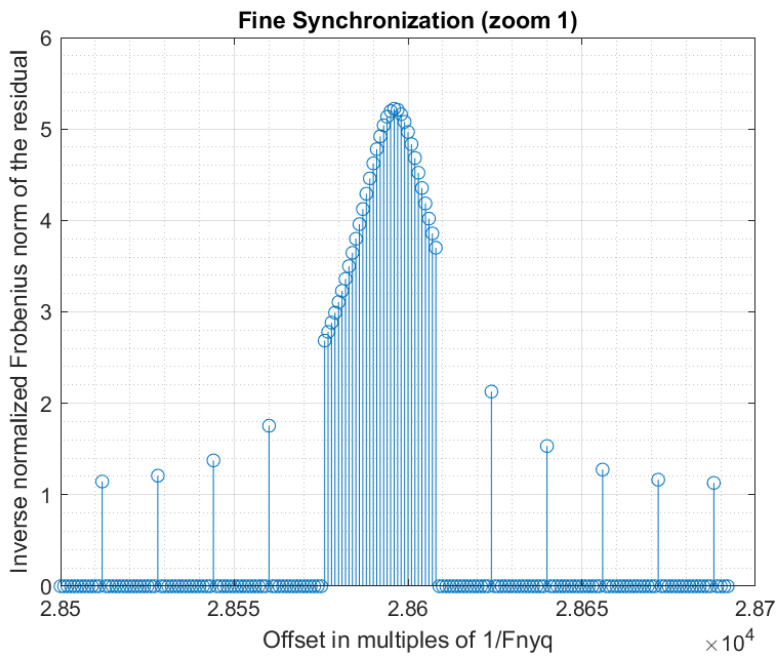
Synchronization (zoom 1).

**Figure 14:**
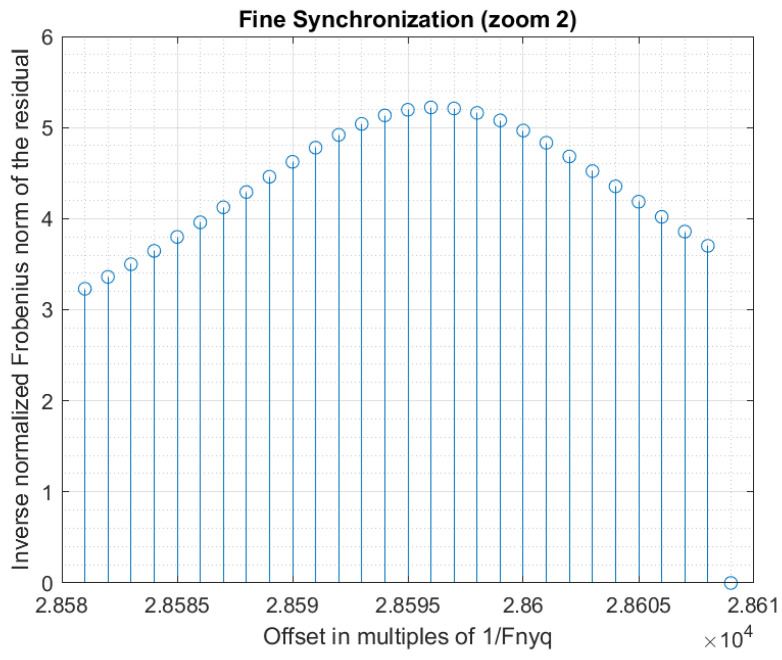
Synchronization (zoom 2).

Using a step g>1 for the evaluation of the synchronization criterion allows a decrease in the computation time by a factor *g*. A good strategy is to obtain a coarse synchronization with a step g>1, and then to perform a fine synchronization around the detected peak. The fine synchronization may even be realized at sub-sample precision, if desired. However, a too large initial step must be avoided because it may lead to missing the synchronization peak during the coarse synchronization. In our experiments, we first used a coarse synchronization with step g=16, then a fine synchronization with step g=1 around the coarse synchronization peak. Figure 12 shows the obtained synchronization data. Figure 13 and Figure 14 present are magnifications of the synchronization peak to show more details.

If the response of the filter is not taken into account in Equation (78) (i.e., assuming an ideal low-pass filter), the synchronization peak is only slightly lower (4.75 instead of 5.22). This is due to the fact that the low-pass filter used in our analog board has good characteristics (almost flat response, and almost linear phase, in the band of interest). The difference would be higher with a lower quality filter. Anyway, it is always better to integrate the filter response in the equations, as we did, because the additional computational cost is negligible.

**Figure 15:**
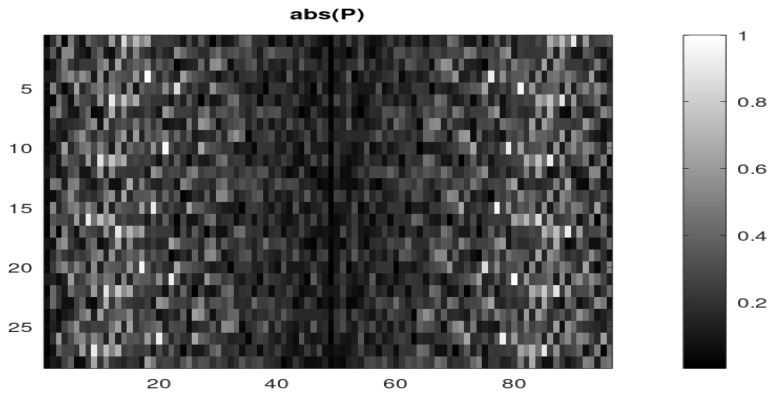
Estimated mixture matrix P (modulus).

Once the signal is synchronized, the estimated matrix $\hat{P}$ is obtained using Equation (104) (at no additional cost because this computation was already part of the synchronization process). Figure 15 shows the modulus of the matrix elements. Here, since we have M=4 physical channels and we have taken q=7, the matrix has qM = 28 rows (and L=96 columns). The first q=7 rows correspond to the first physical channel, then the next *q* rows to the second physical channel, and so on.

**Figure 16:**
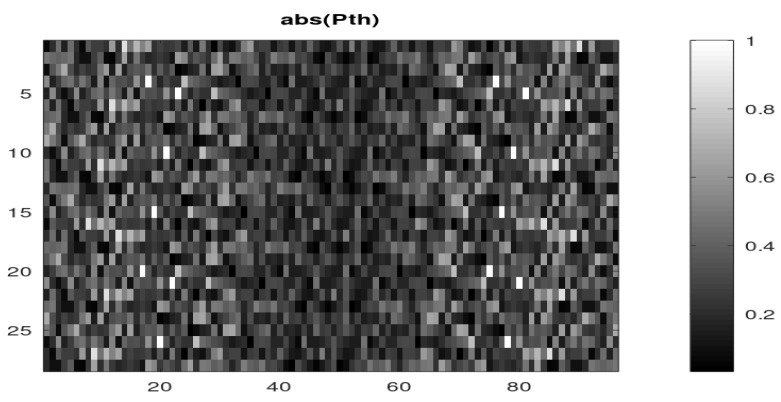
Theoretical mixture matrix $P_{th}$ (modulus).

The theoretical (ideal) matrix $P_{th}$ can be computed using Equation (100). Since the analog scrambling sequence is the output of a Digital to Analog Converter (DAC), fed with a digital pseudo-random sequence, it is (ideally) piecewise constant in the time domain. In the spectral domain, this is equivalent to a multiplication by the sinc function in which first zero is at $F_{nyq}$. We take that into account when computing our theoretical matrix in order to be as close as possible to the real-world matrix. Figure 16 shows the modules of the elements of this matrix.

**Figure 17:**
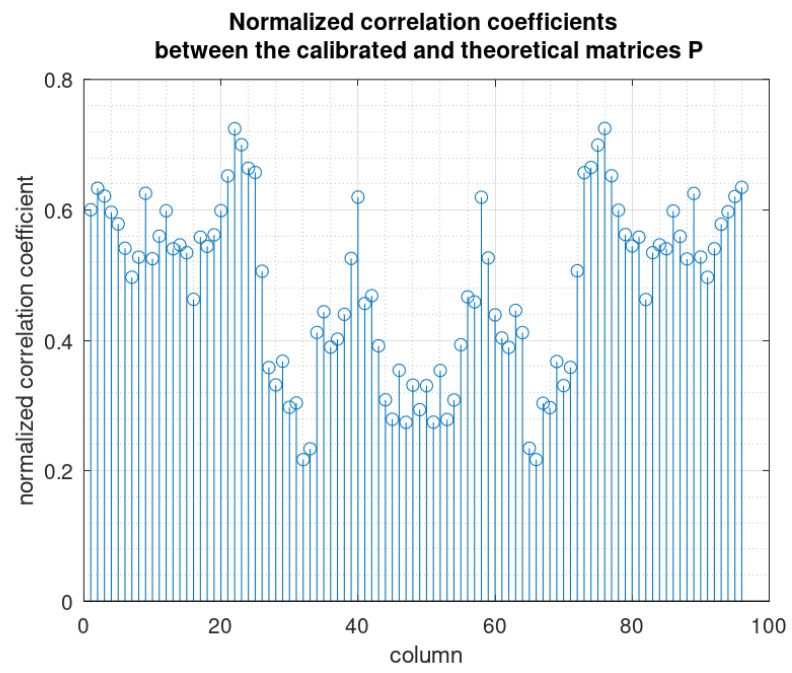
Normalized correlation coefficients.

The estimated (real-world) mixture matrix $\hat{P}$ may be compared with the theoretical (ideal) mixture matrix $P_{th}$. On the basis of the elements modules, we can see that their overall aspects are close despite noticeable differences. In fact, the main differences are in the phases of the elements. If we draw the normalized correlation coefficients between the columns of both matrices (Figure 17), we obtain low values, which confirms significant differences. We remind that a normalized correlation coefficient is the absolute value of the cosine of the angle between two vectors (here the columns of both matrices), then values around 0.5 mean that the angle is about 60 degrees, and thus, the columns are significantly different.

**Figure 18:**
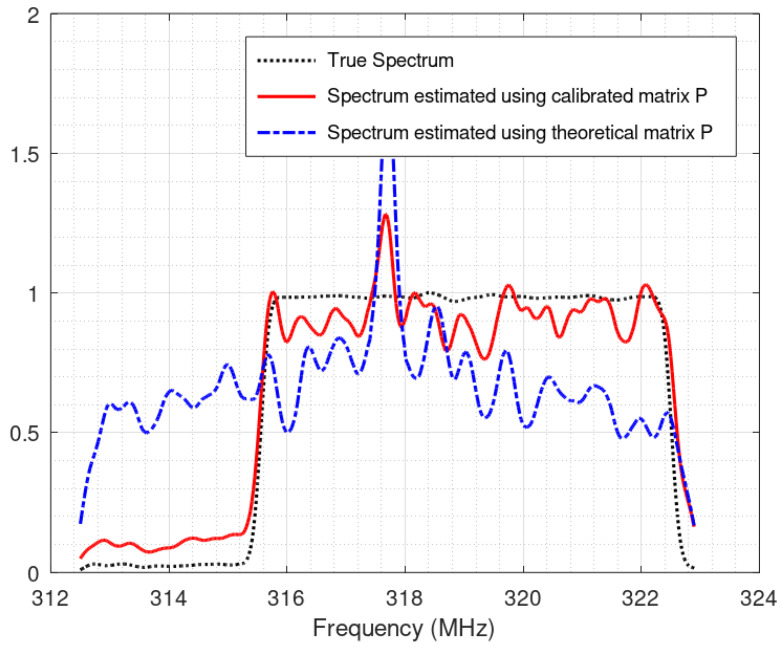
Example of sub-band spectrum reconstruction with and without calibration.

In a previous paper [19], we showed that despite the good quality of our real-world acquisition board, calibration of the system is absolutely required: using the theoretical matrix leads to poor reconstruction performances. Without calibration, the system usually incorrectly detects the active sub-bands, and even when the active sub-bands are correctly identified, the spectrum reconstruction provided by the uncalibrated system is extremely poor, as illustrated in Figure 18.

**Figure 19:**
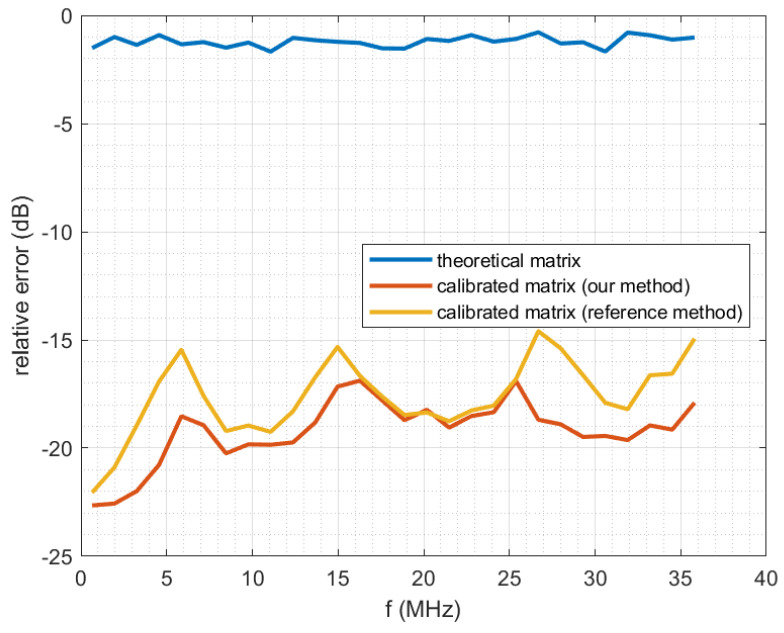
Relative error between computed and observed system output, as a function of output frequency.

Figure 19 shows the relative error between computed and observed system output, as a function of output frequency. The computation is performed using a formula similar to the criterion used in [32].

(133) $$\varepsilon \left(f\right)=20\log_{10}\left(\frac{∥output_{real}\left(f\right)-output_{computed}\left(f\right)∥}{∥output_{real}\left(f\right)∥}\right)$$

Here the frequency band of the acquisition system output signal has been divided into 28 subbands, and $output_{real}$ is the real output signal comprised in the subband centered on *f*. Similarly, $output_{computed}\left(f\right)$ is the computed output signal comprised in the subband centered on *f*, “computed” meaning that it is computed from the input signal using the system model and a given matrix *P*. The three curves correspond to using different matrices *P* in this computation. It can be seen that our method and the reference method provide a good prediction of the real output (relative errors around −18 dB), while the theoretical uncalibrated matrix provides poor results (large relative errors). We remind that in any case, even for the theoretical matrix, we always use a low-pass filter that is calibrated: the true frequency response of the filter (Figure 5 and Figure 6) is being taken into account in the equations through the diagonal matrix $D_{\tilde{h}}$.

**Figure 20:**
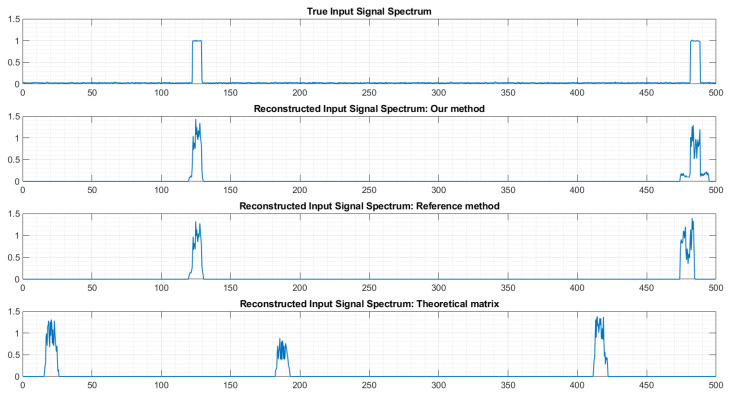
Example of spectrum reconstruction with 2 transmitters. The horizontal axes are frequencies, labeled in MHz.

To further illustrate the interest of the approach, Figure 20 shows the spectrum of the reconstructed signal using different matrices *P*. On top, it is the true spectrum. Here we have two transmitters in the monitored bandwidth. We can see that our method, as well as the reference method [13], correctly identifies the presence of two active transmitters. On the contrary, when using the theoretical matrix, the number of transmitters and their frequency locations are incorrect.

**Figure 21:**
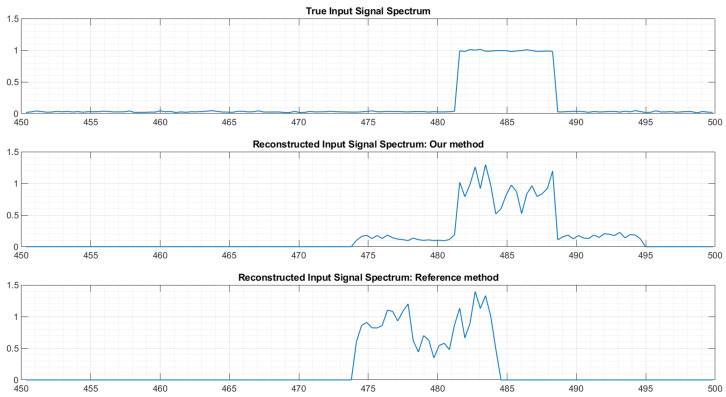
Example of spectrum reconstruction with 2 transmitters (zoom). The horizontal axes are frequencies, labeled in MHz.

If we zoom in on Figure 20, we can see (Figure 21) that the frequency location of the second transmitter is more precise when using matrix *P* provided by our method than when using the matrix provided by the reference method.

**Figure 22:**
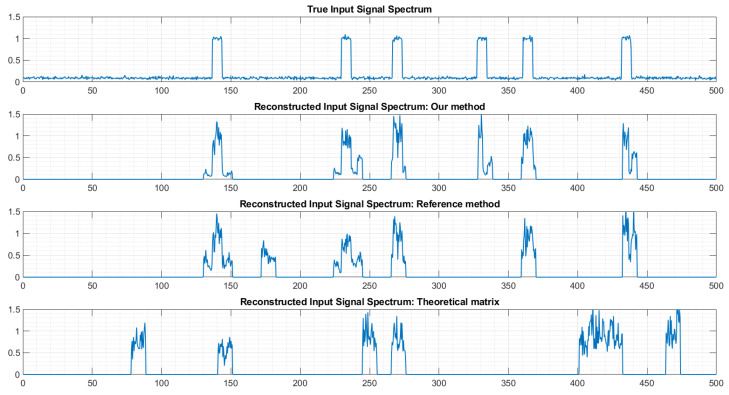
Example of spectrum reconstruction with 6 transmitters. The horizontal axes are frequencies, labeled in MHz.

Let us now consider a much more difficult case, where six transmitters are simultaneously active in the monitored bandwidth (Figure 22). Using the matrix *P* provided by our approach leads to a correct estimation of the number of transmitters and of their frequency location, despite imperfect shapes of the spectrum reconstruction for some transmitters. The reference method produced quite good results also, but missed one transmitter (around 330 MHz) and detected a non-existing transmitter (around 180 MHz). Finally, as anticipated, using the theoretical matrix *P* lead to a reconstruction that is almost totally false.

This shows that calibration is unavoidable. An interest of the extremely fast calibration procedure proposed in this paper is the possibility of performing quick recalibration of the system as soon as the performances appear to decrease. Indeed, many factors, such as temperature, external perturbation, components aging, etc., modify the characteristics of the system, making a recalibration necessary.

## 8. Conclusions

In this paper, we have established an MWC model solely based on linear algebra. It is very convenient as a basis for fast and efficient programming of simulation, reconstruction and calibration algorithms related to MWC. It suppresses a previous restriction on the channels augmentation factor, hence providing more degrees of liberty to the systems designer. It also allowed us to develop an extremely fast implementation of a previously proposed calibration algorithm, leading to a gain of a factor greater than 20 on the computation time. This fast calibration allows quick recalibration of the system as soon as it becomes necessary. Our future work will include more in-depth exploitation of the advantages and interesting properties of this model.

## Figures and Tables

**Table 1:** Parameters of our MWC prototype.

Symbol	Meaning	Value
*M*	Number of physical channels	4
*L*	Length of scramblers	96
$F_{nyq}$	Sampling frequency of scramblers = bandwidth to monitor	1 GHz
$F_{s}$	Sampling frequency of physical ADC	104.1667 MHz
$b=F_{nyq}/F_{s}$	Physical subsampling factor	9.6
$F_{p}=F_{nyq}/L$	Repetition frequency of scramblers	10.41667 MHz

## Data Availability

Not applicable.

## Abbreviations

The following abbreviations are used in this manuscript:

ADC	Analog to Digital Converter
DAC	Digital to Analog Converter
DFT	Discrete Fourier Transform
FFT	Fast Fourier Transform
flop	floating point operation
MWC	Modulated Wideband Converter
SVD	Singular Value Decomposition

## Appendix A. Mathematical Proofs

### Appendix A.1. Frequency-Domain Downsampling Matrix Ξ

Using the general radix identity, with N=ab, the inverse DFT matrix can be decomposed as:

(A1) $$F_{N}^{-1}=P_{a,b}^{-1}\left(I_{a}⊗F_{b}^{-1}\right)T_{a,b}^{-1}\left(F_{a}^{-1}⊗I_{b}\right)$$

Then, the frequency-domain downsampling matrix Ξ is:

(A2) $$\begin{array}{cc} \Xi & =F_{N}^{-1}\left(I_{a}⊗1_{b}^{T}\right)F_{a} \end{array}$$

(A3) $$\begin{array}{cc} \; & =P_{a,b}^{-1}\left(I_{a}⊗F_{b}^{-1}\right)T_{a,b}^{-1}\left(F_{a}^{-1}⊗I_{b}\right)\left(I_{a}⊗1_{b}^{T}\right)F_{a} \end{array}$$

(A4) $$\begin{array}{cc} \; & =P_{a,b}^{-1}\left(I_{a}⊗F_{b}^{-1}\right)T_{a,b}^{-1}\left(F_{a}^{-1}⊗1_{b}^{T}\right)F_{a} \end{array}$$

(A5) $$\begin{array}{cc} \; & =P_{a,b}^{-1}\left(I_{a}⊗F_{b}^{-1}\right)T_{a,b}^{-1}\left(I_{a}⊗1_{b}^{T}\right) \end{array}$$

(A6) $$\begin{array}{cc} \; & =P_{a,b}^{-1}\left(I_{a}⊗F_{b}^{-1}\right)\left(I_{a}⊗1_{b}^{T}\right) \end{array}$$

(A7) $$\begin{array}{cc} \; & =\frac{1}{b}P_{a,b}^{-1}\left(I_{a}⊗\theta_{b}^{T}\right) \end{array}$$

(A8) $$\begin{array}{cc} \; & =\frac{1}{b}\theta_{b}^{T}⊗I_{a} \end{array}$$

### Appendix A.2. Periodic Scrambler

Using the general radix identity, with N=KL, the DFT matrix can be decomposed as:

(A9) $$F_{N}=\left(F_{K}⊗I_{L}\right)T_{K,L}\left(I_{K}⊗F_{L}\right)P_{K,L}$$

Then, we have:

(A10) $$\begin{array}{cc} \begin{array}{cc} & \bar{s} \end{array}\; & =sF_{N} \end{array}$$

(A11) $$\begin{array}{cc} \; & =\left(\theta_{K}⊗p\right)\left(F_{K}⊗I_{L}\right)T_{K,L}\left(I_{K}⊗F_{L}\right)P_{K,L} \end{array}$$

(A12) $$\begin{array}{cc} \; & =\left(\theta_{K}F_{K}\right)⊗\left(pI_{L}\right)T_{K,L}\left(I_{K}⊗F_{L}\right)P_{K,L} \end{array}$$

(A13) $$\begin{array}{cc} \; & =K\left(1_{K}⊗p\right)T_{K,L}\left(I_{K}⊗F_{L}\right)P_{K,L} \end{array}$$

(A14) $$\begin{array}{cc} \; & =K\left(1_{K}⊗p\right)\left(I_{K}⊗F_{L}\right)P_{K,L} \end{array}$$

(A15) $$\begin{array}{cc} \; & =K\left(1_{K}I_{K}\right)⊗\left(pF_{L}\right)P_{K,L} \end{array}$$

(A16) $$\begin{array}{cc} \; & =K\left(1_{K}⊗\bar{p}\right)P_{K,L} \end{array}$$

(A17) $$\begin{array}{cc} \; & =K\bar{p}⊗1_{K} \end{array}$$

### Appendix A.3. Commutation Property of $C_{\bar{x}}^{\left(K\right)}$

(A18) $$\begin{array}{cc} C_{\bar{x}}^{\left(K\right)}J_{N}^{K} & =\left(KI_{L}⊗1_{K}\right)C_{\bar{x}}J_{N}^{K} \end{array}$$

(A19) $$\begin{array}{cc} \; & =K\left(I_{L}⊗1_{K}\right)J_{N}^{K}C_{\bar{x}} \end{array}$$

(A20) $$\begin{array}{cc} \; & =K\left(I_{L}⊗1_{K}\right)\left(J_{L}⊗I_{K}\right)C_{\bar{x}} \end{array}$$

(A21) $$\begin{array}{cc} \; & =K\left(I_{L}J_{L}\right)⊗\left(1_{K}I_{K}\right)C_{\bar{x}} \end{array}$$

(A22) $$\begin{array}{cc} \; & =J_{L}\left(KI_{L}⊗1_{K}\right)C_{\bar{x}} \end{array}$$

(A23) $$\begin{array}{cc} \; & =J_{L}C_{\bar{x}}^{\left(K\right)} \end{array}$$

where we have used Equation (24).
